# Supplementary material for: Modular Assembly of Phosphite Dehydrogenase and Phenylacetone Monooxygenase for Tuning Cofactor Regeneration
Source: Biomolecules. 2021 Jun 17;11(6):905. doi: 10.3390/biom11060905 (PMC8234031; doi:10.3390/biom11060905)
Supplement: Supplementary file 1 [file biomolecules-11-00905-s001.zip › biomolecules-1258656-supplementary.pdf]

Supplementary Data

Modular Assembly of Phosphite Dehydrogenase and Phenylacetone Monooxygenase for Tuning Cofactor Regeneration

Nyoman Purwani[a,b], Caterina Martin[a], Simone Savino[a], and Marco W. Fraaije[a*]

[b]Molecular Enzymology Group, University of Groningen, Nijenborgh 4, 9747 AG Groningen, The Netherlands
[a]Department of Health, Faculty of Vocational Studies, Universitas Airlangga, Kampus C Mulyorejo, Surabaya 60115, Indonesia

[*] Corresponding author. E-mail address: m.w.fraaije@rug.nl

Table S1. Oligonucleotide primer for PTDH and PAMO ORF amplification.

Primer	Primer (direction 5’-3’/ sequence)
PTDH For	CCTGAAGGTCTCGTGGTATGCTGCCGAAACTGGTCATCACCCATC
PTDH Rev	CCCGGTCTCTCACCGTCAGCCGCAGGGTTGGCTTTCG
PAMO for	CCTGAAGGTCTCGTGGTATGGCCGGGCAGACGACTGTC
PAMO rev	CCCGGTCTCTCACCGGTGAGGACGAAACCTTCGTA
PAMO Bsa mut for	CTGCGCTCAGCAACATGCTGGTGTCTATC
PAMO Bsa mut rev	CACGTGCTGTTCGATAGACACCAGCATGTTG

Table S2. Synthetic gene constructs.

Synthetic genes	Base sequences
RIAD	CCTGAAGGTCTCGGGTGGTGGTGGTAGCGGTGGCGGTGGTTCAG
RIAD-RIAD	GCGGCGGTGGTTGCAGAGACCGCTAAAGTTGCGGTCTCTTTGCG
	GTCTGGAACAGTATGCAAATCAGCTGGCAGATCAGATTATCAAA
	GAAGCAACCGAAGGTTGCTAACTTGAGAGACCGGGAATGAAGG
	TCTCGTTGCGGTAGCCTGCGTGAATGTGAACTGTATGTTCAGAAA
	CATAATATTCAGGCCCTGCTGAAAGATAGCATTGTTCAGCTGTGT
	ACCGCACGTCCGGAACGTCCGATGGCATTTCTGCGCGAATATTTT
	GAACGTCTGGAAAAAGAAGAAGCCAAATAACTTGAGAGACCGG
	AC
RIDD	CAAAGGTCTCATTGCGGTCTGGAACAGTATGCAAATCAGCTGGC
	AGATCAGATTATCAAAGAAGCAACCGAAGGTTGCGGCGGTGGTG
	GTAGCGGTGGCGGTGGTTCAGGCGGCGGTGGTTGTGGCCTGGAA
	CAGTACGCGAATCAGCTGGCCGATCAGATCATCAAAGAAGCGAC
	CGAAGGCTGTTAACTTGAGAGACCAATGAAAGACGTTTCGATAT
	GCTATAGCTATTTAGACTATATGGATATGCTATCGATAGATCGAT
	CGGATATCGATTTAAATTCGATTTAAAATTATTATGCTATAGGCT
	ATAG

Table S3. Molecular weights of size exclusion chromatography standards.

Standard Protein	MW	Log MW	Elution Vol (mL)
Thyroglobulin	670000	5.83	9.48
γ-globulin	158000	5.20	12.5
Ovalbumine	44000	4.64	15.0
Myoglobin	17000	4.23	16.82
Vitamin B12	1350	3.13	19.75

Table S4. Conversion of bicyclo[3.2.0]hept-2-en-6-one. Conversion was measured by GC-MS analysis, based on product peak areas.

Enzymes complexes	Time (hours)	Conversion (%)
$PTDH_A$-$PAMO_I$	5	59±4.2
	24	88±0.2
$PTDH_{A2}$-$PAMO_I$	5	60±0.7
	24	94.5±2.6
$PAMO_A$-$PTDH_I$	5	60±5.6
	24	78.5±0.7
$PAMO_{A2}$-$PTDH_I$	5	61.5±0.2
	24	77.1±2.6
PTDH-PAMO	5	68±2.8
	24	94.5±2.1

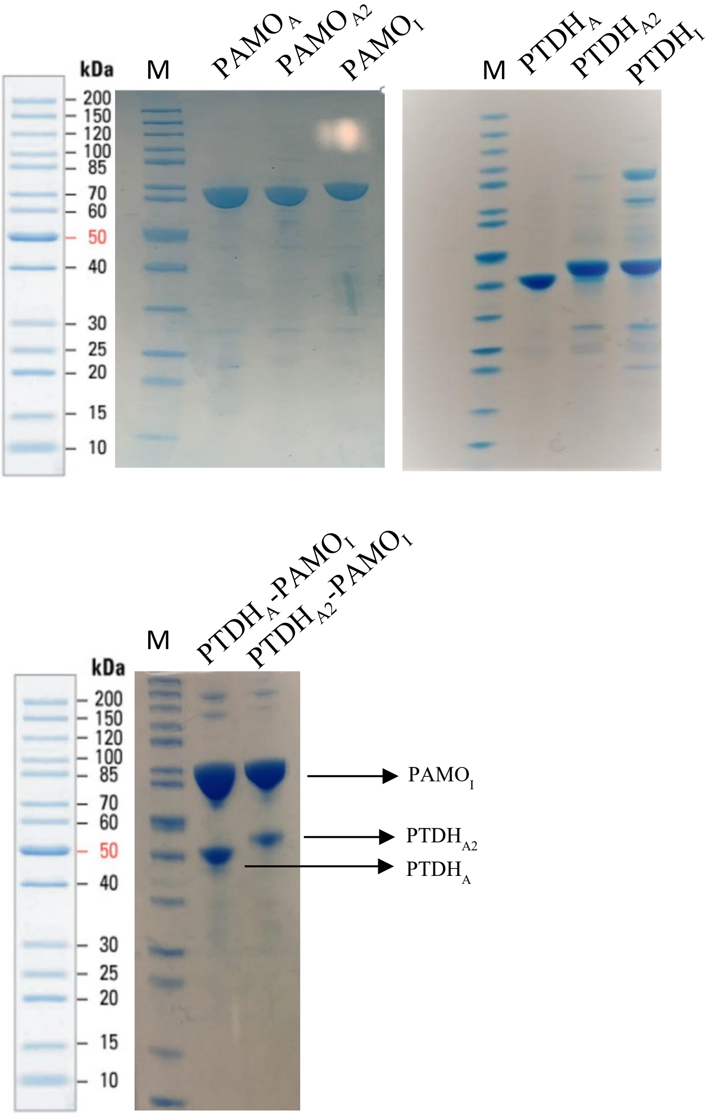

Figure S1. SDS-PAGE analysis. A: analysis of purified tagged PAMO variants; B: analysis of purified tagged PTDH variants; C: complex $PTDH_A$-$PAMO_I$ and $PTDH_A$-$PAMO_I$.

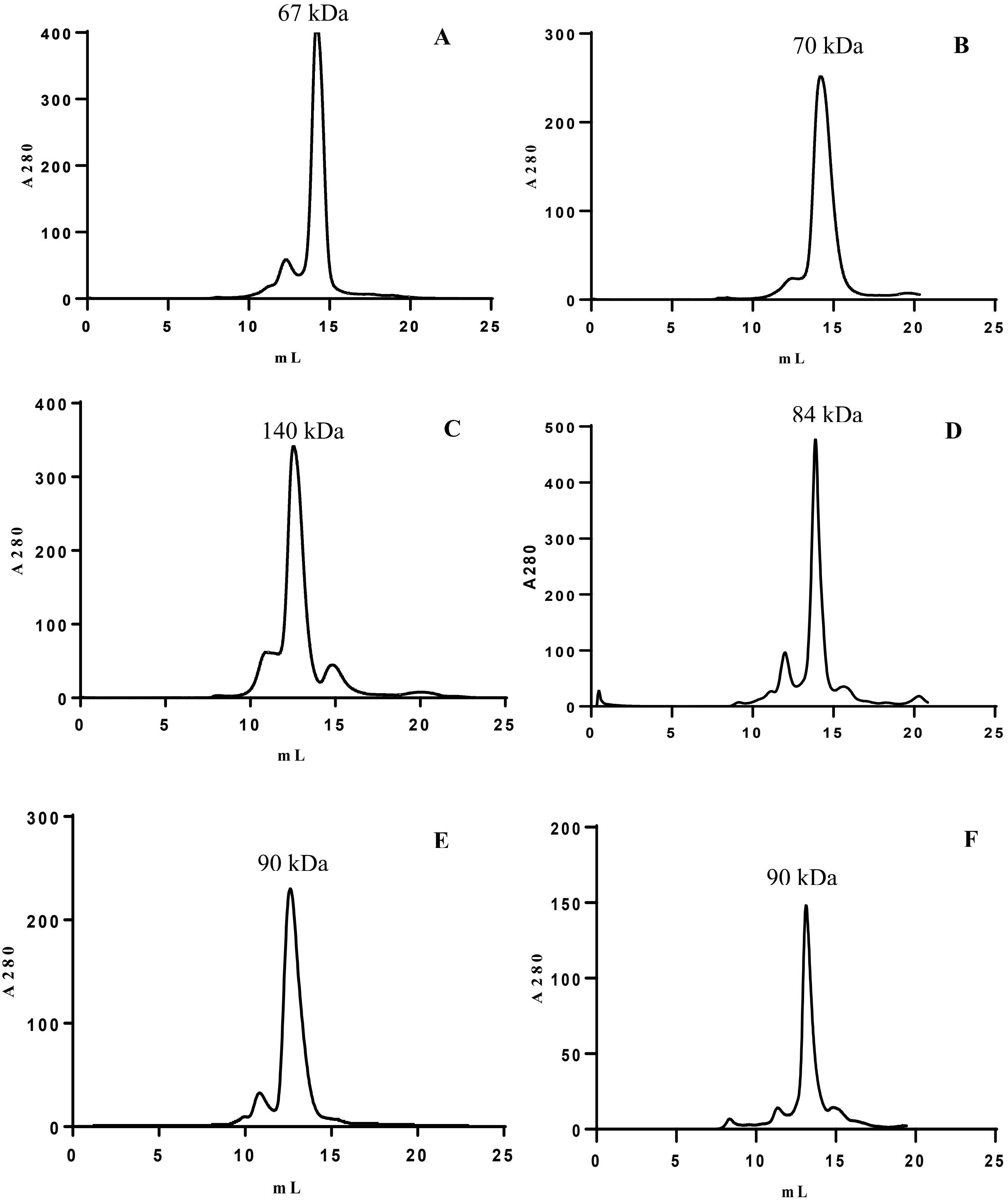

67 kDa
A
70 kDa
B
140 kDa
C
84 kDa
D
E
90 kDa
F
90 kDa
A 280
A280
m L
0
5
10
15
20
25
0
100
200
300
400
500
0
50
150

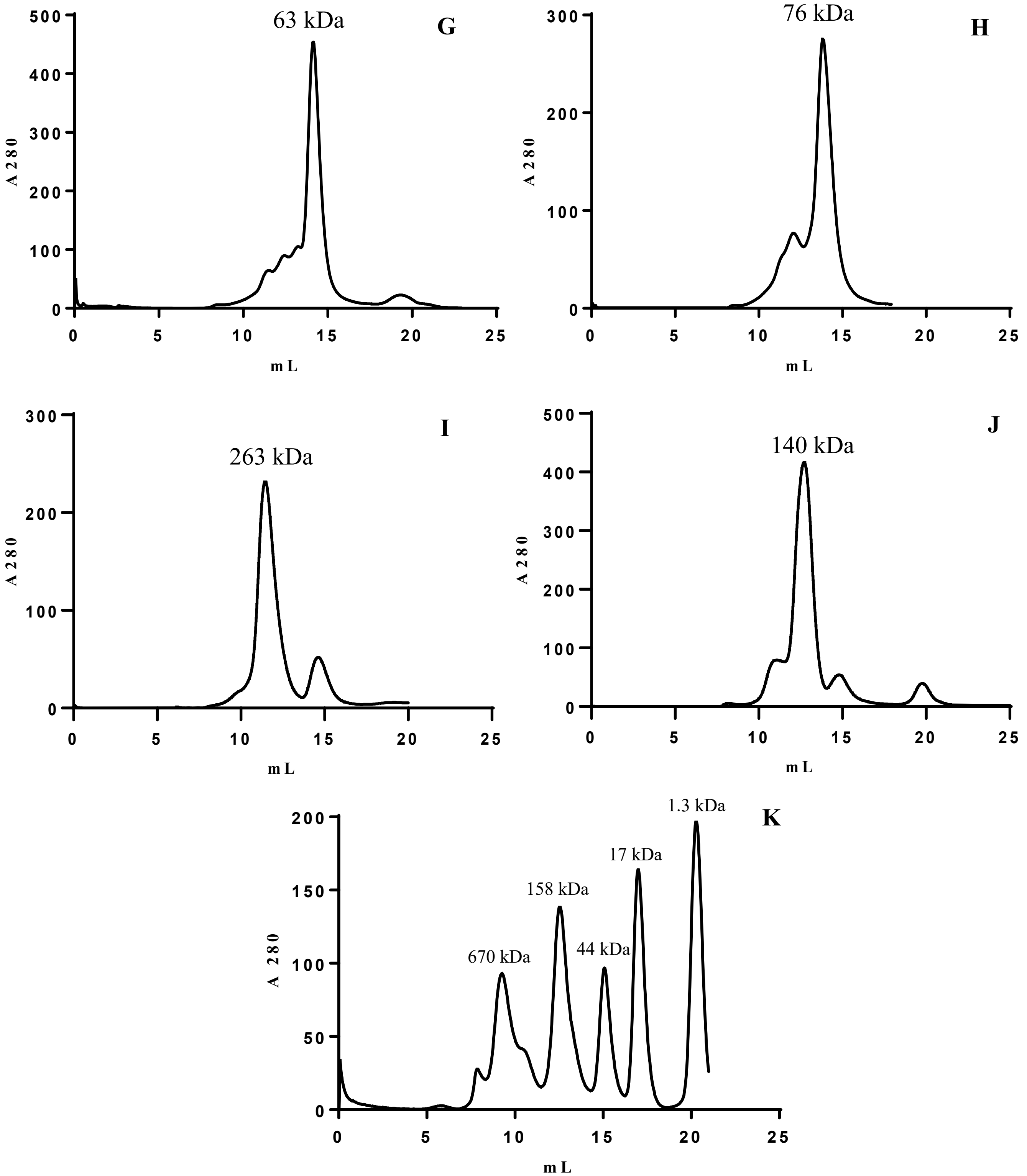

Figure S2. Size exclusion chromatogram of (A) PAMO$_{A}$, (B) PAMO$_{A2}$, (C) PAMO$_{I}$, (D) PTDH$_{A}$, (E) PTDH$_{A2}$, (F) PTDH$_{I}$, (G) PAMO$_{A}$-PTDH$_{I}$, (H) PAMO$_{A2}$-PTDH$_{I}$, (I) PAMO$_{A}$-PAMO$_{I}$, (J) PAMO$_{A2}$-PAMO$_{I}$, (K) size exclusion protein standards.
